# Strategies to Improve the Robustness and Generalizability of Deep Learning Segmentation and Classification in Neuroimaging

**DOI:** 10.3390/biomedinformatics5020020

**Published:** 2025-04-14

**Authors:** Anh T. Tran, Tal Zeevi, Seyedmehdi Payabvash

**Affiliations:** 1 Department of Radiology, Columbia University Irving Medical Center, NewYork-Presbyterian Hospital, Columbia University, New York, NY 10032, USA;; 2 Department of Biomedical Engineering, Yale University, New Haven, CT 06520, USA;

**Keywords:** robustness, generalization, neuroimaging, deep learning, segmentation, classification

## Abstract

Artificial Intelligence (AI) and deep learning models have revolutionized diagnosis, prognostication, and treatment planning by extracting complex patterns from medical images, enabling more accurate, personalized, and timely clinical decisions. Despite its promise, challenges such as image heterogeneity across different centers, variability in acquisition protocols and scanners, and sensitivity to artifacts hinder the reliability and clinical integration of deep learning models. Addressing these issues is critical for ensuring accurate and practical AI-powered neuroimaging applications. We reviewed and summarized the strategies for improving the robustness and generalizability of deep learning models for the segmentation and classification of neuroimages. This review follows a structured protocol, comprehensively searching Google Scholar, PubMed, and Scopus for studies on neuroimaging, task-specific applications, and model attributes. Peer-reviewed, English-language studies on brain imaging were included. The extracted data were analyzed to evaluate the implementation and effectiveness of these techniques. The study identifies key strategies to enhance deep learning in neuroimaging, including regularization, data augmentation, transfer learning, and uncertainty estimation. These approaches address major challenges such as data variability and domain shifts, improving model robustness and ensuring consistent performance across diverse clinical settings. The technical strategies summarized in this review can enhance the robustness and generalizability of deep learning models for segmentation and classification to improve their reliability for real-world clinical practice.

## 1. Introduction

Machine learning and artificial intelligence have revolutionized medical imaging workflows and applications in recent years [1]. These tools are applied before or during image acquisition for purposes such as denoising, radiation dose reduction, image reconstruction, and workflow optimization—including scheduling exams, triaging patients, and prioritizing imaging studies. The downstream applications of artificial intelligence include image analysis, computer-assisted diagnosis, radiology report generation, and clinical decision support. Machine learning models used in medical imaging have shown remarkable potential for improving diagnosis and treatment planning [2], by harnessing imaging patterns that are imperceptible to human eyes. The main downstream applications of these tools in medical image analysis can be categorized into segmentation and classification tasks, which are the focus of this review. However, their successful deployment in clinical practice depends on ensuring consistent performance across diverse real-world scenarios. This challenge highlights the need for models that are both robust to imaging variations and generalizable across different clinical settings.

Robustness refers to a model’s ability to maintain performance despite the variability in medical imaging environments [3]. This variability often arises from multiple sources, including differences in scanner manufacturers and types, scan acquisition protocols, patient positioning within the imaging machine, image artifacts, and noise [4–6]. Without robust models, even minor changes in image quality or acquisition parameters can result in substantial classification errors or imprecise segmentation boundaries [7,8].

Generalizability, on the other hand, extends beyond robustness and focuses on a model’s ability to perform effectively on entirely new, unseen datasets [9–11]. This property is essential for translating research into clinical practice, ensuring reliable performance across diverse patient populations, and maintaining accuracy across healthcare settings. Models lacking generalizability often fail to capture universally essential features, instead relying on spurious correlations in the training data (overfitting), which limits their practical utility in real world scenarios.

To address these challenges, researchers have developed a variety of strategies to enhance both robustness and generalizability. Data augmentation techniques simulate realistic variations in medical image acquisition by applying controlled changes to contrast, resolution, orientation, and noise levels, reflecting differences in imaging protocols and scanner types [12,13]. Adversarial training improves a model’s resilience by exposing it to the potential noise and distortions that are encountered in clinical settings. Transfer learning leverages pre-training on large-scale medical imaging datasets, followed by fine-tuning for specific clinical applications, while domain adaptation minimizes systematic differences between images acquired at different medical centers [14,15]. Inverse supervised learning [16], which complements traditional supervised learning by focusing on the inverse mapping between inputs and outputs, can also reduce overfitting to specific patterns in the dataset and enhances interpretability by highlighting the causal factors behind predictions. These approaches succeeded across various neuroimaging tasks, including tumor detection, brain structure segmentation, and neurocognitive disease classification [17,18], ensuring consistent clinical performance across different clinical settings and patient populations [19,20].

While previous articles have summarized the methods used to increase the robustness and generalizability of machine learning models in general or with a narrow focus on a specific task [21–24], our review provides an overview of strategies for improving the robustness and generalizability of deep learning models in neuroimaging to achieve a balance between accuracy and multi-modal adaptability for clinical applications. In addition, segmentation and classification are core tasks in the downstream application of artificial intelligence tools in neuroimaging. Segmentation requires pixel-level robustness, classification requires feature-level robustness, and they are most strongly affected by domain shifts in neuroimaging. We summarize the previous studies on improving the robustness and generalization ability of deep learning models in neuroimaging segmentation and classification tasks, such as transfer learning, regularization, and adversarial training; we further emphasize the important role of evaluation metrics and uncertainty estimation.

## 2. Methods

This comprehensive review follows a structured protocol [25] to retrieved and summarize the current state of robustness and generalizability in deep learning models for neuroimaging. Our methodology includes a systematic literature search and summarizes various strategies. The review addresses three key aspects of deep learning models in brain imaging:

- The current state and challenges in model robustness and generalizability.
- Strategies for enhancing and monitoring these attributes.
- Barriers in transitioning models from research into clinical practice.

### 2.1. Search Strategy

Our search strategy targeted three major databases: Google Scholar, PubMed, and Scopus. The key search terms were categorized as follows: (i) Primary: “brain images” OR “neuroimaging” OR “brain imaging” OR “neuro-imaging”; (ii) Task-specific: (“segmentation” OR “classification”) AND “deep learning”. (iii) Model-focused: “robustness”, “generalizability”. These terms were systematically combined using Boolean operators to achieve comprehensive yet focused search results.

### 2.2. Selection Criteria

**Figure 1.**
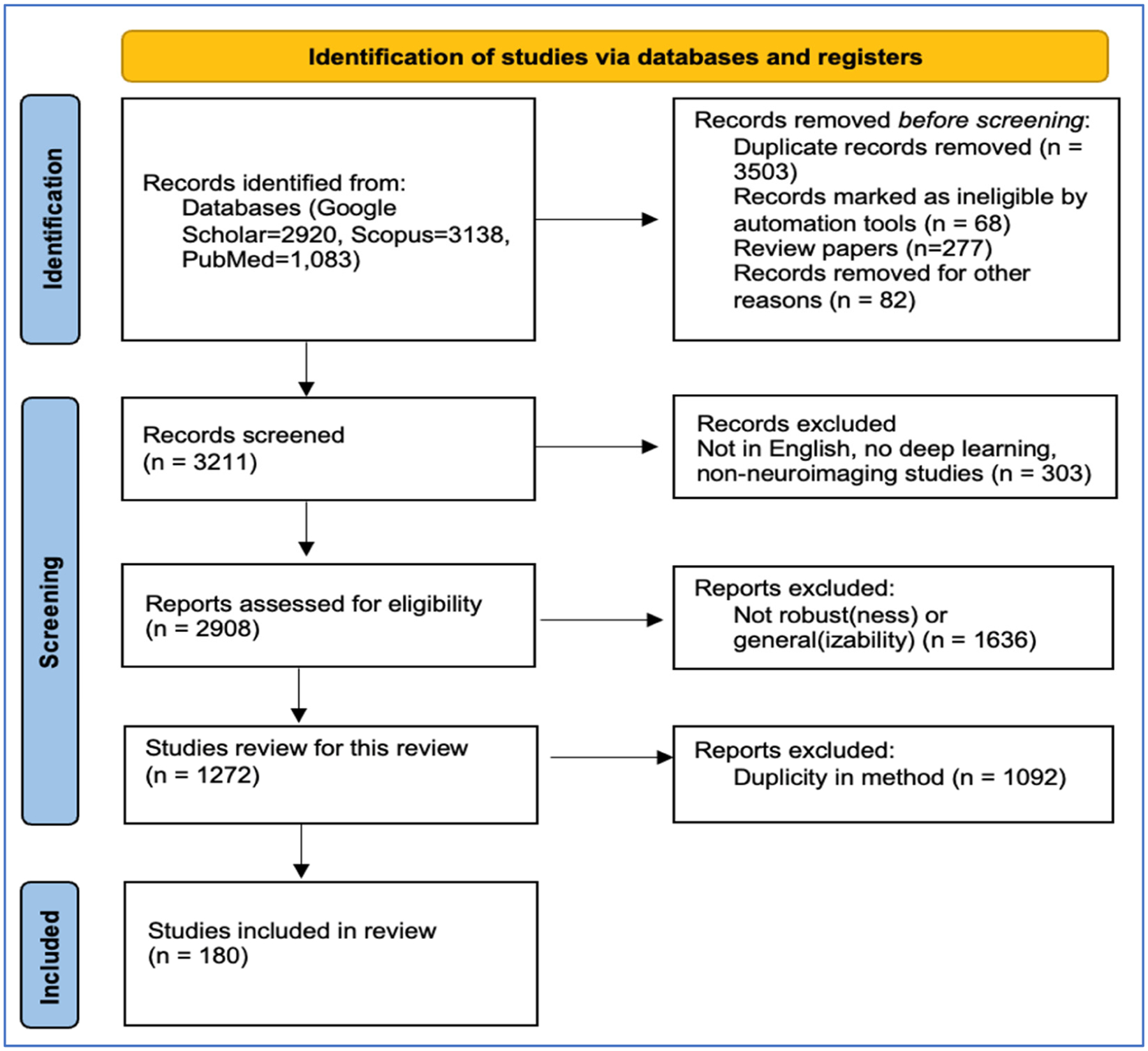
Flowchart of the search and selection of studies.

We included studies that met the following quality and relevance criteria: peer-reviewed, English-language publications with accessible full texts, focusing on original research in neuroimaging. The exclusion criteria were survey studies, duplicate studies, and research not directly addressing model robustness or generalizability. Figure 1 depicts the flowchart of our search strategy and the final number of articles that are referenced in our review. While our study was not conducted as a formal systematic review, we applied PRISMA principles to guide our search strategy [26].

### 2.3. Data Extraction

Relevant information was systematically collected by carefully reviewing each study. The strategies were categorized into three subcategories: definition, training usage, and evaluation methods. The study parameters were organized using a spreadsheet, with details such as the objectives, deep learning models, network architectures, publication year, journal, datasets, performance metrics, and challenges placed in separate columns. Each study was listed as a separate row for clarity.

## 3. Strategies for Improving Robustness and Generalizability

**Figure 2.**
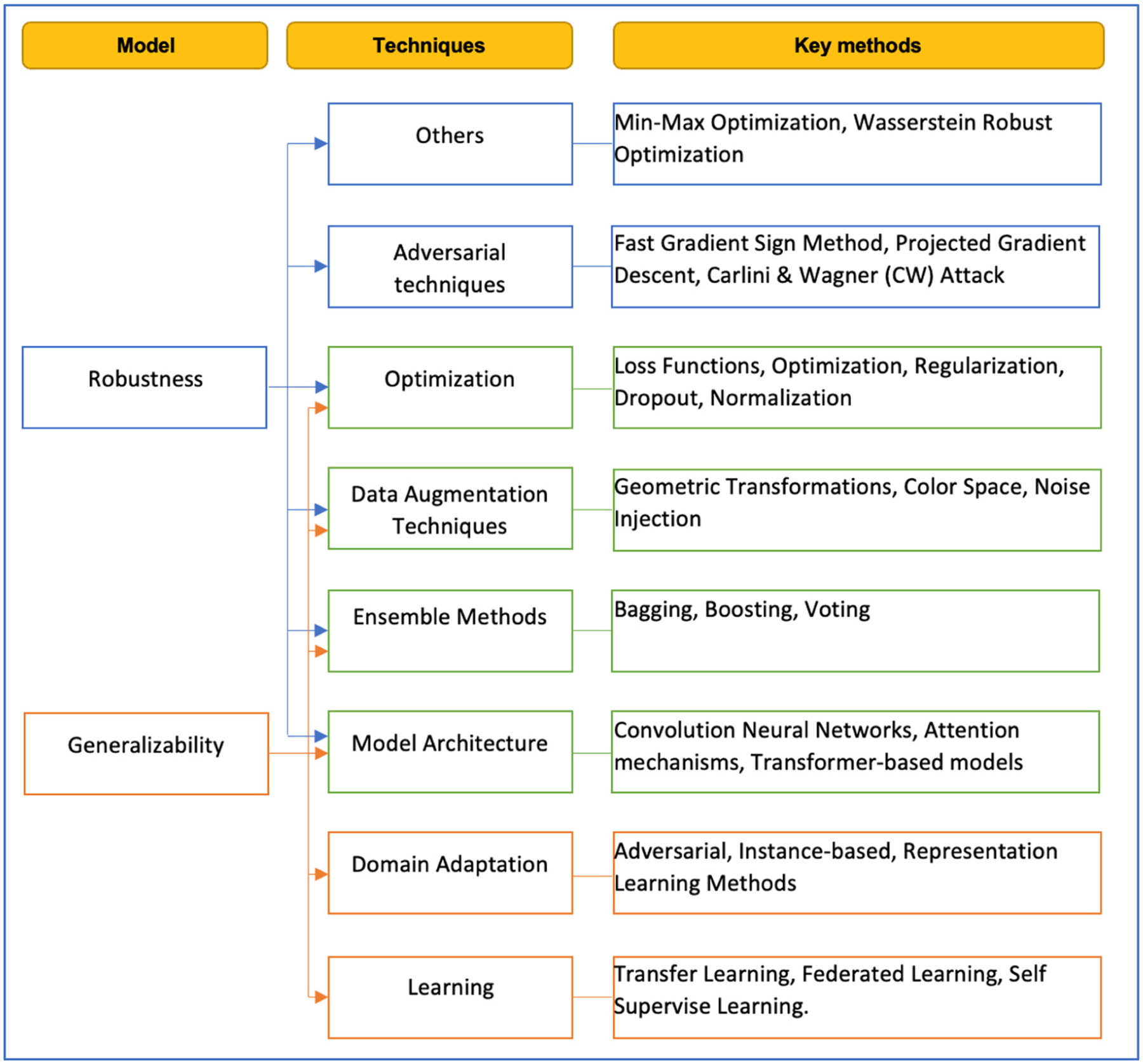
Strategies for improving robustness and generalizability.

Figure 2 summarizes the strategies and key methods for improving the robustness and generalizability of segmentation and classification deep learning models.

### 3.1. Shared Approaches Improving Both Robustness and Generalizability

#### 3.1.1. Optimization Techniques

The loss function [27] quantifies how well the model’s predictions match the ground truth labels and is used during optimization to update the model parameters. Dice loss [28], which is widely used in segmentation tasks, measures the overlap between the predicted segment and the actual segment, ensuring high-quality segmentation, supporting both robustness and generalization. For classification, in the case of imbalanced data, using weighted cross-entropy loss [29] helps the model to focus on underrepresented classes, improving its performance on diverse data.

Adaptive optimization methods: techniques such as Adam [30] dynamically adjust the learning rate to stabilize the training process and improve convergence, especially in noisy or incomplete data.

Regularization helps prevent overfitting by introducing constraints to the learning process, ensuring that models capture general patterns rather than features limited to training data. Several key regularization approaches have been established in the field. L1 Regularization (Lasso) [31] adds penalties based on absolute coefficient values, promoting model sparsity, while L2 Regularization (Ridge) [32] applies penalties based on squared coefficient values to encourage even weight distribution. In neural networks, Dropout [33] randomly deactivates neurons during training to prevent over-reliance on specific pathways, and Batch Normalization [34] normalizes layer inputs to stabilize training to enhance reliability. Early Stopping [35] prevents overfitting by monitoring the validation performance and halting training at the optimal point.

Feature size reduction, or dimensionality reduction, is a crucial step in the preprocessing pipeline for deep learning models in neuroimaging. The most popular techniques using feature reduction in neuroimaging are Principal Component Analysis (PCA) [36], Independent Component Analysis (ICA) [37] and feature selection techniques such as LASSO [38]. The PCA and Autofeat techniques led to increased accuracy for the models in EEG-based emotional state classification [39]. Each technique has its strengths and limitations, and selecting the appropriate method depends on the specific neuroimaging task. Summaries of different feature size reduction strategies and their applications are included in Supplemental Material Section S1.

#### 3.1.2. Data Augmentation

Data augmentation improves model performance by diversifying the training data without the need to collect additional samples [40]. This strategy includes a range of transformation approaches. Geometric transformations involve operations such as rotation, flipping, scaling, and cropping [41,42], while color space augmentation adjusts brightness, contrast, and saturation [43]. Noise injection introduces various types of noise to improve model resilience [44,45], and random erasing selectively occludes regions of an image to mimic real-world variability [46]. Advanced methods such as Mixup and CutMix combine images to create novel training examples, further enriching the training dataset [47].

#### 3.1.3. Ensemble Learning Approaches

Ensemble learning improves model robustness and generalizability by combining multiple models into a stronger predictive system, leveraging the principle that diverse models can collectively overcome individual limitations. Each ensemble technique offers unique advantages for enhancing model reliability in medical imaging applications.

Key ensemble techniques include the following: bagging (Bootstrap Aggregating) [48,49], i.e., the independent training of multiple models on random subsets of data (using bootstrapping), and aggregating predictions through averaging or majority voting; boosting [50,51], which trains models sequentially, with each model focusing on correcting errors made by its predecessors by assigning higher weights to misclassified samples; stacking (Stacked Generalization) [52,53], which trains multiple models on the same dataset, using their predictions as input features for a meta-model that produces the final output; and voting ensembles [54], a technique that combines predictions from multiple models through either majority voting (hard voting) or probability-weighted voting (soft voting).

#### 3.1.4. Model Architecture

Model architecture improvements refer to strategies that enhance the structure and design of machine learning models to improve performance, robustness, and generalization. These improvements often involve changes in the organization of layers, the use of specialized mechanisms, or the introduction of innovative training strategies. U-Net [55] with skip connections is widely used for segmentation tasks in medical images. Variants such as Attention U-Net [56] further improve feature extraction and spatial consistency, enhancing both robustness and generalization. Transformer-based Networks: Vision Transformers (ViT) [57,58] and hybrid Convolutional Neural Network (CNN)-Transformer models [59] are capable of capturing long-range dependencies in brain images, improving both robustness and generalization by focusing on important spatial relationships.

### 3.2. Robustness Improvement Methods

#### 3.2.1. Adversarial Training

Adversarial Training [60–62] enhances model robustness by defending against adversarial attacks, i.e., carefully designed perturbations intended to cause model failure. This approach employs different methods to create adversarial examples of original scans: The Fast Gradient Sign Method (FGSM) [63–65] creates adversarial examples by perturbing inputs along the gradient direction of the loss function. Projected Gradient Descent (PGD) [63–65] extends this by generating stronger adversarial examples through iterative optimization. The Carlini and Wagner (CW) Attack [63–65] iteratively optimizes perturbation that minimizes the perceived change while maximizing the model’s misclassification probability. The effectiveness of these strategies is systematically evaluated using specific attack scenarios and defense efficacy metrics [62,66–68].

#### 3.2.2. Other Methods

Advanced optimization techniques can further improve model robustness. Min–Max Optimization [69] trains models under worst-case scenarios, effectively preparing them for adversarial conditions. Wasserstein Robust Optimization [70] addresses distribution shifts by employing the Wasserstein distance metric in the optimization process. These methods complement traditional approaches by targeting specific vulnerabilities that standard training procedures may not fully address.

### 3.3. Generalizability Improvement Methods

#### 3.3.1. Domain Adaptation and Invariant Learning

Domain Adaptation [71] focuses on improving model performance across different domains or data distributions: it includes Feature alignment and matching the statistical properties of datasets. For example, in brain tumor segmentation, domain adaptation can address variations caused by different MRI protocols. Augmented domain adaptations, such as style transfer methods, further mitigate domain mismatches by transforming source data to mimic target domain characteristics, ensuring robust performance across scanners [72]. In addition, Karthik Gopinath et al. [73] trained neural networks on a vastly diverse array of synthetically generated images with random contrast properties.

Invariant learning emphasizes the extraction of features unaffected by domain-specific variations to ensure consistency across environments. Methods such as Invariant Risk Minimization and causal representation learning eliminate unwanted correlations, focusing instead on causal relationships that generalize well [74]. Contrastive learning has also been applied, particularly in tasks such as stroke lesion segmentation in the brain, by enhancing within-domain similarities and minimizing cross-context differences [75].

#### 3.3.2. Model Training Strategies

Transfer Learning [76,77] enables models to leverage knowledge from one task to improve performance in related tasks. In medical imaging, it is particularly valuable for addressing limited labeled data while maintaining high performance. Key strategies include: feature extraction, where pre-trained models are used to extract relevant imaging characteristics, as demonstrated in brain MRI analysis [78]; fine-tuning, where pre-trained models are adapted to specific tasks through continued training with adjusted learning rates [79,80]; and frozen layers, where early-layer weights are preserved while adapting later layers in neural networks, optimizing computational efficiency and reducing overfitting [81,82]. These methods have proven effective in reducing training times and data requirements [76,83,84].

Federated learning [85] also allows collaborative model training across institutions without the exchange of raw data, ensuring privacy and addressing data governance concerns. By aggregating updates from locally trained models, federated learning can create robust models that are capable of generalizing across varied datasets. Techniques such as federated averaging and differential privacy enable learning from diverse data distributions while safeguarding data privacy [86,87].

Self-supervised learning [88] is a machine learning paradigm that leverages large amounts of unlabeled data to learn useful representations without relying on manual labels. By designing tasks where the dataset itself provides supervision, self-supervised learning enables models to learn underlying patterns and structures that generalize well to many downstream tasks.

### 3.4. Evaluation and Monitoring

Evaluating the effectiveness of robustness and generalization techniques requires comprehensive metrics and systematic monitoring to ensure that models maintain reliable performance across various scenarios, patient populations, and imaging conditions post-deployment.

#### 3.4.1. Key Performance Metrics and Statistical Results

For segmentation tasks, spatial overlap metrics such as Intersection over Union (IoU) and the Dice–Sørensen coefficient (DSC) quantify the accuracy of region delineation across different datasets and conditions [89,90]. Another metric, the Hausdorff distance (HD) [91], measures the maximum distance of a surface set to the nearest point in the other set. These metrics are widely used to assess models’ accuracy and consistency in the presence of anatomical variations and differences in image quality.

Classification tasks are evaluated using complementary metrics. Accuracy provides an overall measure of correct predictions, while Precision and Recall offer insights into model reliability for different classes. The F1 Score, as the harmonic mean of precision and recall, balances these aspects to assess overall robustness. Additionally, the Area Under the Curve of Receiver Operating Characteristic (AUC-ROC) and the Confusion Matrix are especially useful for evaluating model performance across different operating thresholds and class distributions [92–94], making them crucial for assessing generalization across diverse patient populations.

Statistical significance and confidence intervals: several studies report statistical significance and confidence intervals to assess the reliability of their results. Common approaches include paired t-tests [95] or Wilcoxon signed-rank tests [96] to compare the performance of different models, and bootstrap methods [97], which estimate the variability of performance metrics. Results are typically presented with 95% confidence intervals, ensuring that the reported performance metrics are reliable and generalizable across different datasets. The list of assessment metrics is included in Supplemental Material Section S2.

Recently, Suhang You et al. [98] proposed SaRF, a novel method that takes salient information through two self-supervised loss terms during training. It improves sequence classification in terms of the F1 score, AUC, and accuracy (ACC), especially for T1 and post-contrast T1 MRI sequences. Eman Younis et al. [99] presented a novel hybrid approach for improved brain tumor classification by combining CNNs and EfficientNetV2B3 for feature extraction, followed by K-nearest neighbors for classification. Table 1 summarizes the techniques for improving robustness and generalizability in neuroimaging, using key performance metrics from notable studies. This overview helps identify techniques that are suitable for different scenarios based on their previous examples.

#### 3.4.2. Computational Complexity Analysis

Computational complexity analysis is an essential step in evaluating deep learning models, especially in neuroimaging, where large and high-dimensional datasets are prevalent. The goal of computational complexity analysis is to understand the time and space requirements of a model, ensuring that it can handle the scale of neuroimaging data without sacrificing performance or efficiency.

Time complexity refers to the amount of time a deep learning model takes to process a given input. In neuroimaging, inputs typically consist of high-dimensional data such as 3D MRI volumes, 4D fMRI data, or multi-modal imaging. The size and complexity of these inputs can significantly impact the training and inference time of deep learning models. Beside batch size and data augmentation, the time complexity is primarily driven by the network architecture. Based on the architecture’s complexity, computational cost, parameter count, and memory usage, we categorize the deep learning models into three main groups:

- Low-complexity models, such as Multilayer Perceptron and basic CNNs, are suitable for small datasets and simple classification tasks.
- Moderate-complexity models such as ResNet [15] and VAEs [127] balance feature learning efficiency and computational cost.
- High-complexity models such as GANs [128] and ViTs [57] achieve state-of-the-art performance but require high computational resources.

Space complexity refers to the amount of memory, especially high-dimensional data on neuroimaging, that a model requires during training and inference. Some of the key aspects that contribute to space complexity include model parameters, activations, and multimodal data. For example, when using 3D U-Net for volumetric medical image segmentation, handling space complexity is a significant challenge due to the high-dimensional input data. Instead of processing entire 3D scans, 3D U-Net splits large volumetric data into smaller patches (e.g., 64 × 64 × 64 voxels).

There are several optimization strategies that reduce computational complexity in deep learning, such as transfer learning, data parallelism, and model parallelism. By using pre-trained models on similar tasks, transfer learning reduces the need to train a model from scratch, which can be computationally expensive [129]. Fine-tuning a pre-trained model requires fewer resources and can still achieve high performance on neuroimaging tasks. For large-scale models and datasets, parallelism techniques can be employed. Data parallelism involves splitting the data across multiple processors, while model parallelism involves splitting the model across processors [130]. This helps speed up both the training and inference times.

#### 3.4.3. Cross-Validation Strategies

Cross-validation (CV) can provide a systematic method for evaluating model generalizability across different data distributions [27,131]. K-Fold CV divides the dataset into K subsets, using K-1 folds for training and one for validation, rotating through all combinations [132,133]. Stratified K-Fold CV extends K-Fold by maintaining proportions (e.g., pathological conditions or comorbidities) across folds, ensuring balanced evaluation [134,135]. In Leave-One-Out CV, each sample serves as the validation set once, which is particularly useful in small datasets [136]. This approach can be extended to evaluate generalizability across different data sources (e.g., Leave-One-Hospital-Out), to assess robustness to institutional imaging protocol variations [137]. Nested CV incorporates two validation loops, providing unbiased estimates of model performance and hyperparameter optimization [138].

#### 3.4.4. Validation Framework

Comprehensive validation allows for the assessment of model performance across multiple dimensions. When conducting validation with out-of-distribution data and adversarial samples, models are evaluated on their ability to maintain performance under previously unseen variation [7,66,139]. Defense efficacy tests robustness against variations in imaging protocols [62,66–68]. The Augmentation effectiveness is assessed using metrics like the Fréchet Inception Distance and Inception Score [140,141], which evaluate the diversity and realism of augmented samples. Ensemble stability, determined by cross-validation performance variance, reflects consistency across data subsets. Training efficiency metrics evaluate models’ adaptability and feasibility across different clinical settings [76,83,84]. Such multi-faceted validation frameworks ensure a comprehensive evaluation of models’ generalizability and robustness.

### 3.5. Pros and Cons of Different Robustness and Generalizability Improvement Methods

The choice of strategy to improve generalizability and robustness depends on the specific requirements and constraints of neuroimaging applications. Each approach offers unique advantages and limitations that must be carefully considered for clinical deployment. These techniques can be broadly categorized into training-time methods, including regularization and data augmentation techniques which enhance model performance during training, and inference-time approaches, including uncertainty estimation and ensemble techniques that improve reliability and robustness during prediction. Validation strategies, including cross-validation and adversarial testing, also evaluate model performance at the time of inference. Each category involves trade-offs in terms of computational demands, implementation complexity, and effectiveness in improving model robustness and generalizability. Table 1 provides a comprehensive overview of these methods, highlighting their strengths, limitations, and notable applications in neuroimaging tasks. This highlights an important concern regarding the practical implementation of complex AI techniques in neuroimaging, especially for resource-constrained settings. While adversarial training, advanced architectures, and ensemble models have shown promising results in improving model robustness and performance, they often incur increased computational costs. These approaches can require more GPU resources, longer training times, and higher memory consumption, creating barriers for smaller research institutes and clinics that do not have such infrastructure.

To address this concern, users can apply strategies that balance performance with computational efficiency. Techniques such as knowledge distillation, in which a smaller model emulates the behavior of a larger, more complex model, can effectively reduce resource demands while maintaining robust performance. Additionally, methods such as quantization, which compresses model weights to a lower precision, and pruning, which removes redundant network connections, are effective in reducing model size and accelerating inference. Incorporating low-rank approximation or a neural architecture search (NAS) can further optimize model design for efficiency. By integrating these lightweight strategies into the discussion, the authors provide a more comprehensive perspective on practical AI implementations, especially for organizations with limited computational resources.

Recently, Barati et al. [142] evaluated the impact of optimizers and loss functions on brain tumor type prediction accuracy. Their study shows that the Adam optimizer combined with either the Categorical Cross-Entropy (CCE) or Binary Cross-Entropy (BCE) loss function outperforms other combinations. Moreover, Nadam and RMSprop outperform other optimizers. The strengths and limitations of techniques used to improve the model’s robustness and generalizability in neuroimaging are shown in Table 2.

## 4. Challenges in Translating Robust and Generalizable Models to Clinical Settings

Deep learning models for neuroimaging segmentation and classification tasks face unique challenges that affect their reliability and adaptability in clinical practice. These challenges arise from the nature of neuroimaging data, as well as the complexity of clinical environments, including population variability, task-specific demands, and workflow constraints. Below, we discuss these challenges using examples from classification and segmentation tasks, focused on Alzheimer’s disease, traumatic brain injury, stroke, and intracerebral hemorrhage (ICH).

### 4.1. Data Quality and Standardization

Neuroimaging data quality is highly variable due to scanner artifacts, acquisition protocols, and preprocessing methods [157]. For example, artifacts such as patient motion during fMRI or DWI acquisition can cause blurring or misalignment, leading to errors in stroke lesions or ICH segmentation [158]. In Alzheimer’s classification, inconsistent intensity normalization across multi-site T1-weighted MRI datasets can negatively impact feature extraction, such as cortical thickness estimations, degrading model performance [159].

Imbalanced datasets also present significant challenges. For instance, brain aging classification models often favor younger adults due to the scarcity of labeled data for older populations, reducing accuracy in predicting age-related neurodegeneration [160]. Similarly, in ICH segmentation, smaller hemorrhages are frequently underrepresented in the training data, leading to overfitting on larger lesions and poor generalization in subtle cases [161].

Proposed dolutions: robust preprocessing pipelines tailored to specific tasks, such as motion correction for DWI in stroke imaging or intensity harmonization for Alzheimer’s studies, are essential [162]. Addressing data imbalance through oversampling underrepresented cases or generating synthetic data using GANs has shown promise [163]. For instance, GANs have been used to simulate infarct lesions to segment stroke lesions, improving segmentation accuracy in noisy settings [164]. Caihua Wang et al. [165] proposed a hybrid framework consisting of multiple CNNs, and a linear SVM to make robust final predictions from limited data.

### 4.2. Population Variability and Cross-Site Generalization

Deep learning models often struggle to generalize across diverse populations and imaging sites due to domain shifts. For example, Alzheimer’s disease classification models trained on data from a single scanner or region may perform poorly when tested on datasets from other regions, reflecting differences in demographics, genetic factors, or scanner properties [166]. In stroke lesion segmentation, differences in imaging protocols (e.g., different b-values in DWI) across institutions can cause domain mismatches, reducing model accuracy [167].

Proposed solutions: Federated learning frameworks enable training across multiple sites without sharing sensitive patient data, thereby exposing models to a broader demographic and scanner variability while preserving data privacy [87,168]. Transfer learning has also proven effective, allowing models pre-trained on one dataset to adapt to specific conditions, such as ICH or Alzheimer’s progression [169].

### 4.3. Task-Specific Reliability in Segmentation and Classification

Segmentation and classification tasks in neuroimaging pose distinct reliability challenges. In segmentation, accurately delineating small or subtle lesions, such as small hematomas in ICH or small ischemic strokes, remains difficult due to the low contrast between pathological and normal tissue [170]. For classification, models may rely on spurious correlations, such as scanner-specific noise, to predict conditions such as Alzheimer’s disease or brain age [171]. Emergency settings exacerbate these issues, with low-quality scans reducing reliability in stroke segmentation. Moreover, models often fail to generalize to atypical stroke presentations, such as chronic infarcts with diffuse boundaries [172].

Proposed Solutions: Uncertainty-aware frameworks can identify subjects where predictions are less reliable, allowing clinicians to focus on areas of high confidence. For instance, Bayesian neural networks have been applied to ICH segmentation to estimate uncertainty in hemorrhage boundaries [173]. For classification, ensemble methods have reduced reliance on spurious correlations, improving robustness in Alzheimer’s diagnosis across multi-site datasets [174].

## 5. Ablation Study: Robustness and Generalizability of Intracranial Hemorrhage Segmentation and Classification from Non-Contrast Head CT

Intracranial hemorrhage (ICH) is a life-threatening condition, as the accumulation of blood within the brain tissues can increase intracranial pressure, potentially leading to irreversible brain injury or death if not diagnosed and treated quickly. Computed tomography (CT) scans are the gold standard for initial diagnosis, as they provide rapid images of the brain and are highly effective in visualizing acute ICH. The early detection of a hemorrhage, as well as its location and its subtype, is crucial in preventing mortality and morbidity in patients with intracerebral hemorrhage. We evaluated the impact of various strategies on improving the segmentation and classification performance for five types of intracerebral hemorrhage (epidural hemorrhage (EDH), subdural hemorrhage (SDH), subarachnoid hemorrhage (SAH), intraventricular hemorrhage (IVH), and intraparenchymal hemorrhage (IPH)), as shown in Table 3.

To enhance the robustness and generalizability of deep learning models for ICH segmentation and classification, several key strategies have demonstrated significant improvements. Augmentation techniques, such as stochastic rotation, elastic deformation, and noise injection, have improved model generalization by simulating the realistic deformations commonly seen in clinical images. This approach has effectively reduced overfitting and improved performance for rare hemorrhage types such as EDH. Meanwhile, optimization strategies incorporating hybrid loss functions (such as Dice + Focal loss) have enhanced boundary delineation and improved model convergence in imbalanced datasets. Regularization has further stabilized the training process, especially when applied to models trained on CT datasets. Additionally, cross-validation using a 5-layer hierarchical scheme promotes model stability across different imaging centers, improving the detection of rare hemorrhage types by ensuring balanced data representation during training. Ensemble learning techniques improve performance in difficult cases characterized by ambiguous boundaries or subtle hemorrhage patterns, enhancing the model’s resilience to noise and artifacts.

Finally, nnUNet and attention-based models demonstrated superior performance by leveraging all these mechanisms to capture long-range dependencies and spatial contexts. This improved feature aggregation significantly enhanced segmentation accuracy and classification accuracy, especially for complex hemorrhage subtypes such as subarachnoid hemorrhage and intraventricular hemorrhage. Together, these strategies enhance the robustness of the model and ensure improved performance across diverse clinical scenarios.

However, many promising techniques have yet to be deployed. Methods such as adversarial training, which enhances model resilience to perturbations, and domain adaptation, which mitigates performance degradation across different imaging centers and scanner types, have yet to be applied in this context. Similarly, transfer learning—which leverages pretrained models to improve learning efficiency in data-limited situations—and self-supervised learning, which allows models to extract meaningful features from unlabeled data, have yet to be explored for ICH segmentation, and classification tasks. Combining these techniques could provide significant gains in model robustness, particularly for handling data variability, noise, and rare types of hemorrhage. Future research integrating these underutilized strategies could further advance the reliability of AI systems in clinical neuroimaging applications.

## 6. Discussion

Despite significant advances in neuroimaging segmentation and classification tasks undertaken by deep learning models, achieving robustness and generalization across diverse conditions remains a significant challenge. Deep learning models are typically sensitive to variations in input data, such as differences in scanner types, noise, artifacts, and acquisition protocols. This lack of robustness can limit the generalization of these models across different datasets and clinical settings. When models are trained on one dataset, they often fail to perform well on others due to the domain shift. Addressing this heterogeneity requires harmonization techniques and a shift toward federated learning approaches.

The generalization capability of deep learning models is a key challenge, especially in neuroimaging. These models often perform well on training data but struggle when applied to unseen data. In medical applications like brain tissue segmentation or characterization, this is a critical issue, as models need to be reliable across diverse patient populations and data sources. Moreover, the scarcity of labeled medical imaging data exacerbates the issue of generalization, as large, annotated datasets are difficult to acquire.

In classification, neuroimaging datasets often suffer from class imbalances, whereby pathological regions are much smaller than healthy brain tissue. This imbalance poses a challenge for deep learning models, as they can easily be overfitted to the more common classes. Additionally, labeled data for medical segmentation are costly and time-consuming to obtain, which limits the availability of the large datasets necessary for training robust models. One promising direction is the use of self-supervised learning, where models learn useful representations from unlabeled data. In the context of neuroimaging, self-supervised techniques can help to leverage large numbers of unlabeled medical images to improve generalization when the labeled data are limited.

Deep learning models are also susceptible to adversarial attacks, which can compromise their reliability in critical applications. To improve robustness to perturbations and domain shifts, adversarial training techniques are being explored. These methods train models to defend against adversarial attacks or variations in input data, making them more reliable across different clinical scenarios. Combining deep learning models with traditional machine learning approaches or incorporating domain knowledge into the architecture could lead to more reliable and interpretable systems. For example, hybrid systems that combine neural networks with rule-based systems could offer both high accuracy and explainability.

It is notable that inherent differences in image acquisition, resolution, contrast mechanisms, and artifacts significantly influence data quality and standardization between various neuroimaging modalities such as MRI, CT or PET. As a result, strategies for improving the robustness and generalizability of machine learning models may need to be tailored to the specific modality. For instance, MRI data may require harmonization techniques to address variability across scanners and protocols, whereas CT images might demand preprocessing to normalize differences in contrast administration or reconstruction algorithms. Therefore, understanding the modality-specific requirements is critical for developing robust and generalizable models for segmentation and classification tasks in neuroimaging.

In addition, some emerging methods in machine learning and related fields may have untapped potential for neuroimaging applications. For example, retrieval-augmented generation (RAG) [184] is a hybrid machine learning framework that combines retrieval mechanisms with generative models. While RAG is typically applied to natural language processing tasks, its principles can be extended to brain image classification by integrating retrieval mechanisms into the decision-making pipeline. This approach can improve interpretability, generalizability, and accuracy in neuroimaging tasks. Hyperbolic CNN has also shown improved generalizability and robustness compared to CNN [185]. Other methods proposed to improve model performance include exploiting the asymmetries of brain scans to detect pathologies [186,187].

Despite these promising advances, many ethical challenges must be addressed to ensure the responsible use of artificial intelligence in medical imaging during data collection, development, and evaluation. Concerns about patient privacy, informed consent, and data ownership arise during data collection. Neuroimaging data often contain extremely sensitive information, making anonymization important to protect patient identities. Additionally, ensuring diverse and representative datasets is essential to preventing algorithmic bias, which may affect certain demographic groups. Informed consent procedures must also clarify how the data will be used, especially in cases where data uses may extend beyond the scope of the original study. During model development and evaluation, ethical concerns include data annotation integrity, model transparency, and performance fairness. Inconsistent labeling or informed annotation practices can reduce model generalizability. Developers must employ validation strategies to ensure that models perform reliably across diverse populations and clinical contexts. Furthermore, interpretability techniques such as SHAP, Grad-CAM, and feature attribution methods should be incorporated to enhance model interpretability, particularly for clinical decision making. Addressing these ethical challenges is key to ensuring that deep-learning-driven neuroimaging solutions are both effective and consistent with patient trust and societal benefit.

Future advances in neuroimaging depend on the development of transparent, reliable, and adaptable algorithms to ensure their integration into clinical workflows. Robust models must prioritize interpretability, allowing clinicians to trust and effectively use these tools. Equally important is adapting these algorithms to diverse patient populations, ensuring equitable healthcare outcomes. Multimodal imaging data fusion, combining insights from multiple imaging techniques, offers a path to significantly improving diagnostic accuracy and providing a more comprehensive understanding of neurological conditions.

To address challenges such as data privacy and heterogeneity, federated and distributed learning frameworks are becoming increasingly important. These approaches enable models to collaboratively learn from decentralized datasets without sharing sensitive information, ensuring data security while leveraging diverse sources. Furthermore, fostering collaboration between researchers, clinicians, and industry stakeholders is essential to linking technological advances to real-world needs. By addressing these challenges and leveraging collective expertise, neuroimaging can evolve into a more efficient, equitable, and patient-centered specialty.

One of the key challenges in evaluating and comparing various strategies aimed at improving robustness and generalizability is the variability in input datasets, performance metrics, and the specific tasks being addressed. These inconsistencies limit effective systematic reviews or and the undertaking of meta-analyses. Therefore, in this article, we provided a comprehensive survey of the literature, highlighting notable approaches (Table 1) and summarizing their respective advantages and limitations (Table 2). Finally, our inclusion and exclusion criteria for articles may introduce bias in this review by limiting the scope to neuroimaging studies; meanwhile, many strategies applied to other body parts may also be translated to brain scans.

## 7. Conclusions

Robustness and generalizability in neuroimaging segmentation and classification are important challenges that directly impact the trustworthiness, reliability, and clinical applicability of these techniques. This review highlights a range of approaches to improving model performance in diverse and unpredictable real-world scenarios. However, several obstacles remain, including the computationally demanding nature of these strategies, the need for the continuous monitoring of model performance in real-world settings, and the evolving nature of medical images, especially MRI sequences. By addressing these challenges and fostering interdisciplinary innovation, the field can move closer to realizing robust and generalizable neuroimaging tools with broad clinical impact.

## Supplementary Material

Supplementary Material

## Figures and Tables

**Table 1.** Examples of the main strategies used to improve robustness and generalizability in neuroimaging segmentation and classification using common performance metrics.

Techniques	Studies	Dataset	Performance	Conclusion
Loss Function [27]	Brain hemorrhage (ICH), intraventricular extension (IVH), and peripheral edema (PHE) segmentation [100].	Huashan Hospital, Fudan University	DSC = 0.92, 0.79, 0.71 and Sen = 0.93, 0.88, 0.81 for ICH, IVH, PHE in segmentation tasks.	DSC loss is essential for segmentation.
	ICH, IVH, PHE segmentation from non-contrast CT [101].	TICH-2	Improved average DSC by 0.02	Focal loss is valuable for class imbalance.
Regularization (L1/L2, Dropout) [31–33]	Regularized feature learning improves MRI sequence classification [98].	Swiss-First study	Improvement in mean accuracy by 4.4% (from 0.935 to 0.976), mean AUC by 1.2% (from 0.9851 to 0.9968), and mean F1-score by 20.5% (from 0.767 to 0.924).	Regularization is critical for training and improves robustness.
	Input-level dropout model for brain metastases segmentation [102].	Oslo University Hospital and Stanford	Improve DSC (0.795 ± 0.104 vs. 0.774 ± 0.104, p = 0.017), and IoU (0.561 ± 0.225 vs. 0.492 ± 0.186, p < 0.001). Tested on 6 datasets.	
Batch Normalization [34]	Convolutional neural network with batch normalization for glioma and stroke lesion detection using MRI [103].	BRATS 2013, 2014, 2015, 2016, 2017 and ISLES 2015.	Improves model convergence and boosts 0.9778 Acc, 0.9754 DSC, 0.9770 Spec, 0.9789 Sen on BRATS dataset 2017	Dependence on batch size Can increase computational cost but help models achieve higher accuracy and generalization.
	the combination of convolution, batch normalization and ReLU activation enhances the network's ability to discriminate and capture relevant information [104]	Kaggle (Brain Tumor MRI Dataset)	Improves with an accuracy of 99.88%	
Data Augmentation [40]	Data Augmentation improve Tumor Classification Using MRI Images [99].	Tianjin Medical University General, Nan Fang Hospital, BR35H	Improvement in precision = 0.9951, recall = 0.9947, F1-score = 0.9944, spec = 0.9977.	Essential for improving robustness, especially in limited datasets.
	StyleGANv2-ADA is proposed for augmenting brain MRI slices [105]	Gazi University Faculty of Medicine, BR35H	BraTS 2021 = 75.18%, and Gazi Brains 2020 datasets = 99.36%, BR35H dataset= 98.99%	
Ensemble Methods [48,49]	Enhancing brain tumor classification through ensemble attention mechanism [106].	BraTS 2019	Improves acc = 0.9894, rrecision = 0.9891, recall = 0.9893, F1-Score = 0.9891, AUC = 0.984	Effective in improving model reliability for classification and segmentation tasks.
	An optimized triplanar (2.5D) model ensemble to generate accurate segmentation with fewer parameters [107]	BraTS 2020	Improving Dice with enhancing tumor = 0.713, whole tumor = 0.873, and tumor core = 0.778	
Model Architecture Improvements	DeeplabV3 + Bayesian optimization for segmentation and classification of brain tumor in MRI scans [108].	Brats 2021	Improves acc = 97.0%, recall = 0.966, spec = 0.988, F1-Score = 0.96, precision = 0.966	Advanced architectures such as SwinUNETR and GNNs can improve performance but have a high computational demand.
	Swin transformers for semantic segmentation of brain tumors [109].	BRATS 2021	DSC and HD in this approach are better than nnU-Net, SegResNet, TransBTS.	
Adversarial Training [60–62]	Robust influence-based training methods for noisy brain MRI [110]	BRATS 2017	Increases robustness, ACC = 89.52 ± 2.61	Effective for improving robustness but computationally intensive.
	Improving robustness in predicting hematoma expansion [111]	ATACH-2, YALE	AUC = 0.8 is the same but increases robustness	
Domain Adaptation [71]	Improving the whole-brain neural decoding of fMRI with domain adaptation [112]	OpenfMRI	The best Acc improvement is 10.47% (from 77.26% to 87.73%)	Highly recommended for multi-site datasets with distribution shifts.
	An unsupervised domain adaptation segmentation model is trained across modalities and diseases [113]	Decathlon medical segmentation challenge, RSNA	+11.55% DSC	
Transfer Learning [76,77]	Transfer learning for accurate brain tumor detection [80]	Brain tumor dataset. Figshare	Highest acc of 99.75%	Worth implementing for tasks with limited labeled data, especially in classification.
	Classification of Alzheimer's disease using DenseNet-201 based on deep transfer learning techniques [114]	AD5C dataset	Acc = 98.24	
Federated Learning [85]	Integrated approach of federated learning with transfer learning for the classification and diagnosis of brain tumors on MRI [115]	Figshare, Br35H, SARTAJ	High precision (0.99 for glioma, 0.95 for meningioma, 1.00 for no tumor, and 0.98 for pituitary), recall, and F1-scores in classification, outperforming existing methods.	Promising multi-institutional collaborations, balancing performance and privacy.
	Enhancing Alzheimer's disease classification through split federated learning [116]	Kaggle	Acc = 84.53%	
Self-Supervised Learning [88]	Improves the performance of classification in task-based functional MRI [117].	Human Connectome Project	Acc improves to 80.2 ± 4.7%	Reliable but heavily reliant on large, labeled datasets.
	Contrastive self-supervised learning for neurodegenerative disorder classification [118]	Alzheimer's Disease Neuroimaging Initiative (ADNI), Australian Imaging, Biomarker and Lifestyle Flagship Study of Aging (AIBL), Frontotemporal Lobar Degeneration Neuroimaging Initiative (FTLDNI)	For AD vs. CN, acc= 82% test subset and acc = 80% independent holdout dataset	

Details of datasets are included in the Supplementary Table S1 [98,99,102,105,119–126].

**Table 2.** Overview of the strengths and limitations of techniques used to improve the model’s robustness and generalizability in neuroimaging.

Technique	Strengths	Limitations	Implementation Considerations	Examples
Loss function (for example, Dice loss)	Often used for segmentation tasks by directly optimizing the overlap (e.g., the Dice coefficient) between the predicted mask and the ground-truth.	Less sensitive to small structures	Used in conjunction with other losses such as cross entropy for better performance on imbalanced datasets.	[142,143]
Regularization (L1/L2/Dropout)	Controls model complexity Reduces overfitting Computationally efficient	Uniform penalty across features May oversimplify important patterns Hyperparameter sensitivity	Balance with domain-specific constraints Considers anatomical priors	[144,145]
Batch Normalization	Stabilizes training Reduces internal covariate shifts Enables higher learning rates	Batch size dependency Memory requirements Inference stability issues	Consider batch size constraints Address multi-site variations	[146,147]
Data Augmentation	Increases effective dataset size Improves generalization Addresses class imbalance	May introduce unrealistic variations Risk of violating anatomical constraints Computational overhead during training	Ensures clinically plausible transformations Validates augmented samples with experts	[148,149]
Ensemble Methods	Robust predictions Uncertainty quantification Handles different aspects of data	Increased computational cost Storage requirements Inference time overhead	Balances diversity and accuracy Considers clinical time constraints	[143,150]
Model architecture improvements	Improved feature extraction: advanced architectures combining CNNs and transformer-based models capture complex patterns in neuroimaging data. Scalability: Modularly designed architectures (e.g., nnU-Net) adapt to different neuroimaging modalities (e.g., MRI, fMRI, PET) Multimodal processing: Models such as multimodal CNNs integrate different types of neuroimaging data, improving robustness Better temporal modeling: attention-based or periodic components efficiently process temporal neuroimaging data such as fMRI and EEG	Increased computational demands, especially for architectures such as transformers and deep CNNs. Potential for overfitting when dealing with small datasets, as seen in neuroimaging. Complex hyperparameter tuning is required for architectures such as attention mechanisms	For segmentation tasks, architectures such as U-Net and its variants (3D U-Net, nnU-Net) are specifically designed for volumetric neuroimaging data Considers Graph Neural Networks (GNNs) for connectivity studies, as they model relationships between brain regions. Uses self-supervised pretraining with architectures like Vision Transformers (ViT) to improve performance on limited labeled data Uses model ensembling or dropout models to reduce overfitting and improve generalization	[109,143]
Adversarial Training	Improves robustness to perturbations Handles image artifacts Better generalization	Computationally intensive May reduce standard accuracy Complex hyperparameter tuning	Use clinically relevant perturbations Balance robustness and accuracy	[151,152]
Domain Adaptation	Addresses scanner variations Handles protocol differences Improves cross-site generalization	Requires data from target domain May not capture all domain shifts Complex implementation	Validates on multiple scanner types Considers temporal domain shifts	[153,154]
Transfer Learning	Leverages knowledge from larger datasets Reduces required training data Accelerates convergence	Source-target domain mismatch can degrade performance May preserve unwanted biases from source domain Requires careful layer-specific fine-tuning	Validates anatomical consistency Adjusts learning rates per layer based on domain similarity	[155,156]

The strengths and limitations of each strategy with representative work are summarized and cited.

The strengths and limitations of each strategy with representative work are summarized and cited.

**Table 3.** A review of the robustness and generalizability of ICH segmentation and classification from non-contrast head CT.

Authors	Dataset	Results	Augmentation	Optimization	Cross-Validation	Ensemble Learning	Model Architectures
Segmentation (Dice as the main accuracy metric)							
Murat Yüce [175]	1508 CTs (QURE500+ RSNA 2019)	IPH = 0.59; IVH = 0.47; EDH = 0.35; SAH = 0.24; SDH = 0.34	✔	✔	✔		nnUNet
Zhegao Piao [176]	82.636 CTs, test 20%	IPH = 0.809; IVH = 0.742; EDH = 0.777; SAH = 0.545; SDH = 0.709	✔	✔			HarDNet based transformer
Chia Shuo Chang [177]	51 CTs, test 14.5%	IPH = 0.924; IVH = 0.858; EDH = 0.816; SAH = 0.567; SDH = 0.82	✔	✔			All Attention U-NET
Mayidili Nijiati [178]	1157 CTs, test 200 CTs	IPH = 0.784; IVH = 0.680; EDH = 0.359; SAH = 0.337; SDH = 0.534	✔	✔			Sym-TransNet
Julia Kiewitz [179]	73 CTs, test 20 CTs	IPH = 0.743; IVH = 0.750; SAH = 0.686; SDH = 0.758	✔	✔	✔		nnUnet
Biao Wu [180]	192 CTs BHSD	IPH = 0.54; IVH = 0.51; EDH = 0.48; SAH = 0.215; SDH = 0.1523	✔	✔	✔		nnUnet
Classification (AUC as main outcome accuracy metric)							
Muhammad Asif [181]	13,334 CTs (CQ500 + RSNA), test 30%	IPH = 0.979; IVH = 0.977; EDH = 0.980; SAH = 0.976; SDH = 0.974	✔	✔			Res-Inc-LGBM
Snekhalatha Umapathy [182]	133,709 slices (CQ500 + RSNA), test 14,600 slices	IPH = 0.99; IVH = 0.98; EDH = 0.99; SAH = 0.99; SDH = 0.99	✔	✔		✔	SE-ResNeXT, LSTM
Shanu Nizarudeen [183]	CQ500, 10%	IPH = 0.98; IVH = 0.98; EDH = 0.96; SAH = 0.98; SDH = 0.98	✔	✔			Attention-based RaNet

## Abbreviations

AI: Artificial intelligence
ViT: Vision transformers
CNN: Convolutional neural network
FGSM: Fast gradient sign method
PGD: Projected gradient descent
CW: Carlini and Wagner
CT: Computed tomography
MRI: Magnetic resonance imaging
fMRI: Functional magnetic resonance imaging
DWI: Diffusion-weighted imaging
IoU: Intersection over Union
DSC: Dice–Sørensen coefficient
HD: Hausdorff distance
AUC-ROC: Area under the curve of receiver operating characteristic
ICH: Intracerebral hemorrhage
IVH: Intraventricular hemorrhage
PHE: Perihematomal edema
Sen: Sensitivity
Acc: Accuracy
Spec: Specificity
BraTS: International brain tumor segmentation
SwinUNETR: Swin UNEt TRansformers
CV: Cross-validation
