# Supplementary material for: Strategies to Improve the Robustness and Generalizability of Deep Learning Segmentation and Classification in Neuroimaging
Source: BioMedInformatics. Author manuscript; Available in PMC 2025 Jun 1. (PMC12014193; doi:10.3390/biomedinformatics5020020)
Supplement: Supplementary Material [file NIHMS2074363-supplement-Supplementary_Material.docx]

**Supplementary materials**

1. **Feature size reduction techniques**

Feature size reduction, or dimensionality reduction, is a crucial step in the preprocessing pipeline for deep learning models in neuroimaging. Neuroimaging data, such as MRI, fMRI, or PET scans, are typically high-dimensional, meaning they consist of a vast number of features (e.g., pixels or voxels in 2D or 3D space). Directly feeding such high-dimensional data into deep learning models can lead to several challenges, including high computational costs, risk of overfitting, and difficulty in generalization. Feature size reduction techniques aim to mitigate these issues by transforming the data into a lower-dimensional space while retaining the essential information needed for accurate predictions. In this section, we explore several feature size reduction techniques commonly used in neuroimaging to optimize deep learning model performance.

- 1. *Principal Component Analysis (PCA)*

Principal Component Analysis (PCA) is one of the most widely used linear dimensionality reduction techniques. It transforms high-dimensional data into a set of orthogonal components, called principal components, that capture the most significant variance in the data. By projecting the data onto a smaller number of principal components, PCA reduces the dimensionality while preserving the most important features.

- Application in Neuroimaging: In neuroimaging, PCA is often applied to voxel-based data, such as MRI or fMRI volumes, to reduce the number of voxels used in the analysis. PCA can identify patterns of brain activity or structure that explain the greatest variance across subjects, enabling more efficient downstream analysis and modeling.
- Benefits: PCA is computationally efficient and straightforward to implement. It helps mitigate the curse of dimensionality by focusing on the most informative features, thereby reducing the computational burden.
- Limitations: PCA is a linear technique, meaning it may fail to capture complex non-linear relationships in the data. Additionally, it assumes that the largest variance corresponds to the most meaningful features, which may not always hold true in the context of neuroimaging.
  1. *Independent Component Analysis (ICA)*

Independent Component Analysis (ICA) is another technique used for feature size reduction, particularly in the context of neuroimaging. Unlike PCA, which focuses on capturing variance, ICA aims to decompose the data into statistically independent components. These components are often more interpretable and can correspond to specific brain networks or functional activations in the case of fMRI data.

- Application in Neuroimaging: ICA is commonly used in resting-state fMRI data to identify distinct brain networks (e.g., the default mode network, visual network, etc.). It can also be applied to preprocess EEG or MEG data to separate signal sources, which is valuable for identifying specific brain activity patterns.
- Benefits: ICA can provide more meaningful and interpretable features, especially when dealing with complex data like brain activity patterns, as it identifies independent sources of information rather than just focusing on variance.
- Limitations: ICA can be computationally intensive, especially when dealing with very high-dimensional data. Additionally, the interpretation of independent components may not always be straightforward, and it may require domain knowledge to identify the most relevant components.
  1. *Feature Selection*

Feature selection techniques aim to identify and retain only the most relevant features from a large set of candidates. Unlike dimensionality reduction methods, which transform features into new spaces, feature selection methods directly eliminate irrelevant or redundant features. Common feature selection techniques include:

- Filter Methods: These methods assess the relevance of individual features based on statistical criteria, such as correlation with the target variable or variance across samples. Features that fail to meet a predefined threshold are discarded. Examples include mutual information, correlation coefficients, or statistical tests like t-tests or ANOVA.
- Wrapper Methods: Wrapper methods evaluate subsets of features by training a model and assessing its performance. Techniques like recursive feature elimination (RFE) iteratively remove the least important features and select the subset that maximizes model performance.
- Embedded Methods: These methods perform feature selection during model training. For example, decision tree-based algorithms like Random Forests or LASSO regression can automatically identify and weigh features during the training process.
- Application in Neuroimaging: In neuroimaging, feature selection can be applied to reduce the number of voxels, channels (for EEG), or regions of interest (ROIs) in brain images. This is particularly useful when dealing with large datasets, such as multi-modal or multi-subject datasets.
- Benefits: Feature selection can improve model interpretability and reduce overfitting by removing irrelevant or noisy features. It can also enhance computational efficiency by reducing the input dimensionality without losing critical information.
- Limitations: Feature selection methods, particularly wrapper methods, can be computationally expensive. They may also require careful tuning of parameters to avoid overfitting or underfitting.
  1. *Autoencoders*

Autoencoders are a type of unsupervised deep learning model that can be used for feature size reduction. An autoencoder consists of an encoder, which compresses the input data into a lower-dimensional representation, and a decoder, which reconstructs the input data from this compressed representation. The encoder learns to retain the most important features while discarding irrelevant information.

- Application in Neuroimaging: Autoencoders have been successfully applied to various neuroimaging tasks, including fMRI data compression, voxel reduction in MRI scans, and multi-modal data fusion. By training an autoencoder on neuroimaging data, the encoder can learn to generate a compressed feature representation that retains essential brain features.
- Benefits: Autoencoders are capable of capturing non-linear relationships between features, making them more flexible than linear methods like PCA. They also provide a way to reduce feature size while preserving important data characteristics.
- Limitations: Autoencoders require large amounts of data for training and can be computationally expensive. Additionally, the quality of the feature reduction depends on the architecture and hyperparameters of the autoencoder, which may need extensive tuning.
  1. *T-Distributed Stochastic Neighbor Embedding (t-SNE)*

t-SNE is a non-linear dimensionality reduction technique commonly used for visualizing high-dimensional data in lower-dimensional spaces (typically 2D or 3D). It is particularly useful for preserving the local structure of the data, making it suitable for exploring complex relationships in neuroimaging datasets.

- Application in Neuroimaging: t-SNE is often used for visualizing the results of clustering or classification tasks in neuroimaging. It helps to reveal patterns or subgroups in the data, such as identifying different brain regions or patient subtypes in fMRI or PET data.
- Benefits: t-SNE is particularly useful for data visualization, helping researchers identify hidden structures in high-dimensional neuroimaging data. It is well-suited for exploratory analysis and understanding complex relationships.
- Limitations: t-SNE is computationally intensive and not well-suited for large-scale datasets. Additionally, it is a non-linear technique, so it may not always preserve global structures or be suitable for direct use in predictive modeling.

**Conclusion**: Feature size reduction techniques are crucial for handling the high-dimensional nature of neuroimaging data. Methods like PCA, ICA, feature selection, autoencoders, and t-SNE help to improve computational efficiency, reduce the risk of overfitting, and enhance model interpretability. Each technique has its strengths and limitations, and selecting the appropriate method depends on the specific neuroimaging task, the type of data being used, and the desired balance between computational efficiency and model performance.

1. **Statistical assessment metrics**
   1. *Segmentation*

Dice Similarity Coefficient (DSC) measures the volumetric overlap between segmentation results and ground truth. Dice is computed where A is the set of foreground voxels in the ground truth and B is the corresponding set of foreground voxels in the segmentation result.

$$Dice= \frac{2(A\cap B)}{\left| A \right|+ \left| B \right|} (1)$$

Hausdorff distance (HD) [91] which measures surface distance is the maximum distance of a set to the nearest point in the other set defined as

$d_{H}\left( X,Y \right)=\max\left\{ d_{\mathrm{XY}},d_{\mathrm{YX}} \right\}=\max\left\{ \max_{x\in X} \min_{y\in Y} d\left( x,y \right),\max_{y\in Y} \min_{x\in X} d\left( x,y \right), \right\} (2)$

- 1. *Prediction*

Accuracy is useful when classes are balanced

$$Acc= \frac{TP+TN}{TP+TN+FP+FN} (3)$$

Binary classifiers’ performance in imbalance data is routinely evaluated with Area Under the Curve (AUC) in Receiver Operating Characteristics (ROC) plots [92], sensitivity, and specificity.

Recall, also called sensitivity, is the proportion of true positives among all positives, and varying between 0 and 1.

$Sensitivity=Recall= \frac{\mathrm{TP}}{TP+FN} (4)$

Specificity measures the proportion of true negatives that are correctly identified by the model.

$$Specificity= \frac{\mathrm{TN}}{TN+FP} (5)$$

where True Positive (TP), True Negative (TN), False Positive (FP), and False Negative (FN).

**Supplementary Table S1. List of datasets used in studies cited in the article**

| **Name** | **Access** | **Images** | **Cases** | **Description** |
| --- | --- | --- | --- | --- |
| The International Brain Tumor Segmentation (BraTS) 2012->2024 [119] | Public | Head native (T1), post-contrast T1-weighted (T1Gd), T2-weighted (T2), and T2 Fluid Attenuated Inversion Recovery (T2-FLAIR) | 335->4500 | The International Brain Tumor Segmentation (BraTS) challenge has focused on the generation of a benchmarking environment and dataset for the delineation of adult brain gliomas. |
| ADNI [120] | Public | Head MRI, fMRI, PET | >1000 | Alzheimer’s disease and neuroimaging research. It provides a comprehensive collection of multi-modal data, including structural and functional imaging, biomarkers, genetic data, and clinical assessments. |
| SINO [121],  part of this dataset was used in the RSNA | Public | Head CT | >9000 | This dataset contains over 9,000 head CT scans, each labeled as normal or abnormal. Each scan contains a reconstructed image (stored in our institution’s PACS and saved as DICOMs) and a corresponding sinogram. |
| Ischemic Stroke Lesion Segmentation (ISLS) 2015- 2022 [122] | Public | Head T1w, T2w, FLAIR, ADC, and DWI | ~400 | multicenter MRI dataset for segmentation of acute to subacute stroke lesions |
| Kaggle (Brain Tumor MRI Dataset) [123] including 3 datasets: figshare, SARTAJ, Br35H | Public | MRI | 7023 (2D images) | This dataset contains 7023 images of human brain MRI images which are classified into 4 classes: glioma - meningioma - no tumor and pituitary. |
| CQ500 [125] | Public | CT | 491 | The CQ500 dataset consists of a total of 491 CT scans, including 205 with ICHs, 40 fractures, 65 middle shifts, 127 mass effects, and 54 normal controls |
| Swiss-First study [98] | Private | DWI, FLAIR, SWI, T1, T1Gd, T2 | 350 | The selected volumes contain both epileptic and healthy image volumes |
| Tianjin Medical University General and Nan Fang Hospital [99] | Private | MRI | 3064 | three types of brain tumors, namely gliomas, meningiomas, and pituitary tumors |
| Tranexamic acid for hyperacute primary IntraCerebral Haemorrhage 2 (TICH-2) trial [125] | Private | CT | 1732 | The non-contrast CT baseline scans were collected from 124 participating centers while complying with the local protocol. With a minimum requirement of axial image orientation, CT scans acquired by using any scanner manufacturer, settings, or thickness were included |
| Oslo University Hospital and Stanford Hospital [102] | Private | MRI | 165 | The patient cohort consisted of a total of 165 patients with brain metastases, enrolled from two different hospitals |
| Gazi University Faculty of Medicine [105] | Private | T1-weighted, T1CE (Gadolinium), T2-weighted, and FLAIR MR | 100 | 50 patients being healthy and 50 patients diagnosed with High-Grade Glioma (HGG). |
| Antihypertensive Treatment of Acute Cerebral Hemorrhage II (ATACH-2) trial [126] | Private | CT | 1000 | ATACH-2 was a multicenter randomized trial that enrolled patients with acute supratentorial ICH within 4.5 h of onset, baseline hematoma < 60 mL, and systolic blood pressure elevation (>180 mm Hg) |
